# Beyond the Metronome: Auditory Events and Music May Afford More than Just Interval Durations as Gait Cues in Parkinson's Disease

**DOI:** 10.3389/fnins.2016.00272

**Published:** 2016-06-14

**Authors:** Matthew W. M. Rodger, Cathy M. Craig

**Affiliations:** School of Psychology, Queen's University BelfastBelfast, UK

**Keywords:** Parkinson's disease, auditory cues, gait, music, affordances

## Introduction

Among the most apparent and adverse symptoms of Parkinson's disease (PD) are disturbances in gait. These include shuffling (small amplitude steps), instability (asymmetry and variability between steps), freezing of gait (cessation of movement and difficulty with initiation), and general disfluencies in walking movements and posture (Morris et al., 1996; Bloem et al., 2004; Grabli et al., 2012). Limitations of pharmacological interventions to alleviate gait disturbances (Lord et al., 2011), have led to interest in exploring non-pharmacological means of enhancing walking in PD, to complement drugs-based approaches. Sensory cueing, in which perceptual guides for movement are presented visually, acoustically, or haptically, is one such approach. While sensory cueing, in particular rhythmic auditory cueing, is a viable and promising approach to enhancing gait in PD, it is our opinion that this approach could be expanded by developing a more action-focussed framework for understanding the information available to patients in sound (cues) and how this information influences gait.

## Auditory cueing as interval-specification

One form of sensory cueing for gait is to present a person with rhythmic sound, such as a metronome or beat-based music, and ask them to walk in time with the sound. This Rhythmic Auditory Stimulation (RAS) as gait cues for PD has been shown to lead to improvements in different aspects of gait, such as step length, duration, speed, and variability (e.g., Thaut et al., 1996; De Icco et al., 2015). An explanation for these improvements is that the cerebellar-thalamocortical circuits in the brain that support detection and synchronization to regular perceptual events are relatively preserved in PD, whereas the basal ganglia-thalamocotrical network that supports actions to one's own internal beat are impaired by the disease (Dalla Bella et al., 2015). Because the timing for action is externalized in RAS, each movement (step) can be matched to each perceptual cue (beat), resulting in a more stable gait pattern with larger steps (Nombela et al., 2013).

**Figure 1:**
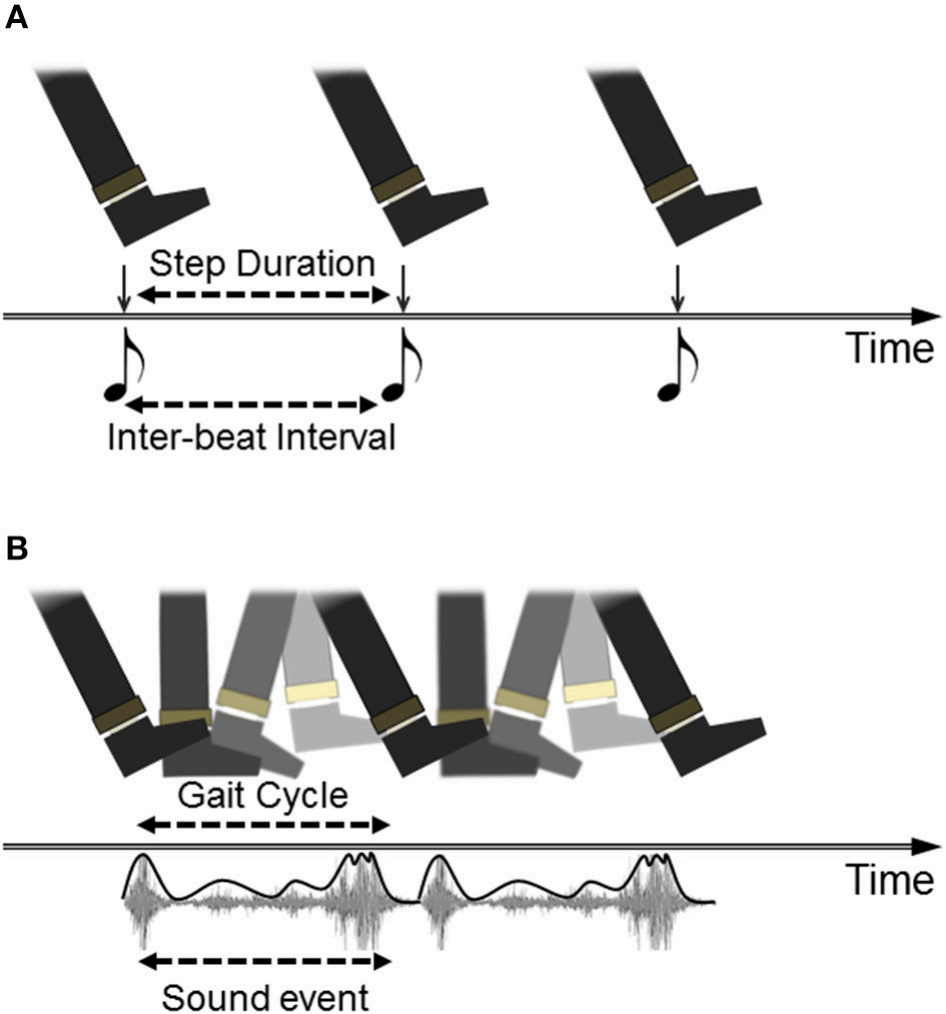
**Different models of auditory cueing of gait. (A)** Walking to a beat is depicted as discrete movement-to-discrete sound mapping. The task for participants (often implicitly instructed) is to temporally match a particular instance within the gait cycle (i.e., heel contact) to the onset of each beat, whether represented by a metronome or by the down-beat of musical rhythm. **(B)** walking to a repeated sound is depicted as the coupling of the gait cycle to dynamic information in the auditory event, in this case the continuous sound made by footsteps on gravel, with the intensity envelope of the sound highlighted in the representation.

Although RAS has shown promising positive effects on gait in PD, this approach seems predicated on a limited view of how auditory events can specify information for action. According to the model, inherited from research on sensorimotor synchronization (SMS) more generally, successfully cued performance is defined as the adjustment of intervals between movement boundaries such that each movement begins/ends at the same moment in time as the onset of the perceptual event (beat). A paradigm of this is finger-tapping to a metronome: accurate tapping is achieved when successive tap events (minimum vertical displacement of the finger) coincide temporally with beat events (peak intensity of sound onset). The translation of this to RAS cueing of gait is represented in Figure 1A. Importantly, this view supports the use of a metronomic (isochronous) beat as the simplest (purest) depiction of interval durations; a listener must simply hop between evenly-spaced islands of sound in a sea of silence (Rodger and Craig, 2014). This model is highly problematic for PD patients, who struggle with synchronizing movements to discrete auditory cues, especially as the disease progresses (Bieñkiewicz and Craig, 2015). Although comparison of synchronization performance to discrete versus continuous sounds has not been directly tested in PD (to our knowledge), recent evidence has shown that continuous sounds lead to better step rhythmicity in patients than a metronome (Young et al., 2016). Hence the idealization of gait cueing as SMS may place an insurmountable burden on PD patients, and indeed is likely an unrealistic picture of what actually occurs during RAS gait cueing, given that most reported research is vague about how participants are instructed to “walk to the sounds” and generally analyse effects on global measures of gait (e.g., average step cadence) rather than step-to-beat synchronization.

## From intervals to events: considering the action-relevance of sounds

The interpretation of RAS gait-cuing as discrete interval-matching not only implies a challenging demand for PD patients, it also draws a limited picture of sensorimotor behavior. Temporal experience is not discretized, but rather is a continuous unfolding flow, populated by meaningful happenings (Gibson, 1975). Moreover, our actions are sequenced, prospectively aimed and ongoing in continuous lived experience (Dewey, 1896; Gibson and Pick, 2000; Merleau-Ponty, 2002). These philosophical concerns have practical consequences: by focusing on cues purely as representations of intervals we may overlook information relevant for action within the perceptual events themselves. In SMS, repeated sounds with identical interval durations can have substantially different effects on synchronized movement timing and trajectory, depending on whether the sound events are discrete or continuous in nature (Rodger and Craig, 2011, 2014). In particular, discrete sounds lead to greater accuracy in coinciding movement end-points with interval onsets, while continuous sounds lead to greater consistency in movement durations and smoother trajectories. These findings can be understood from the perspective of the mappings afforded between sensory and movement events—discrete sounds specify clear event boundaries to map movement boundaries onto, while continuous sounds provide information throughout the movement to couple onto and produce regular repetitive actions, but which are not anchored as tightly to pre-set interval boundaries. This description frames auditory cues for gait as *affordances*, defined as opportunities for action specified in the sensory array, detectable by an agent in its environment (Gibson, 1979). In thinking of cueing sounds as auditory affordances, one must take seriously the way the event is specified in perception, the action-possibilities afforded in the event, and the capabilities of the perceiver to detect and act upon such an event (Steenson and Rodger, 2015).

An illustration of how auditory cues can be considered as action-specifying events is provided by research using footstep sounds as gait cues. Although rhythmic sounds can be artificially generated to be as simple as possible, such as short-tone metronomes, many rhythmic sounds in our environments are more richly patterned by the actions which produce them. Examples include the slosh of oars through water when rowing, the clunks of a chain gang working on a rail line, and the sounds made by footsteps during walking. As listeners, we are particularly tuned into the actions (and actors) that make such sounds and can make accurate judgments about these (Li et al., 1991; Kennel et al., 2014). From footstep sounds, listeners can recognize the gender or emotion of the walker (Li et al., 1991; Giordano and Bresin, 2006), and can discern the spatial properties of the walking action, including step length (Young et al., 2013). Furthermore, listeners can make use of spatial properties of footstep sounds in their own actions. Young et al. (2013) showed that when asked to walk as though they were generating the sounds of recorded footsteps, participants spontaneously adjusted their own step-length in accordance with the step lengths that produced the recorded sounds. Hence, walking sounds influenced spatial as well as temporal properties of gait in listeners. This is represented in Figure 1B as a repeating but continuous movement event (foot plantar rotation across the ground) mapped to a continuous auditory event (gravel crunch). Importantly for present purposes, this effect was also found for people with PD: walking to the sounds of long strides on gravel led to increases in patients' step length (Young et al., 2014). Interestingly, these effects were comparable to a condition in which participants were asked to walk with long steps in time to a metronome, but the gravel footsteps led to the added benefits of reduced variability in both step length and duration. Hence, the action-relevant gravel sounds specified parameters of walking beyond merely step-to-step intervals which was useful in guiding stepping movements in people with PD.

## Music as richly-structured, action-relevant sound events

One rich source of action-relevant auditory events is music. A number of research programmes investigating auditory cueing in PD have used music as RAS, either pre-recorded songs with patient-tailored tempo adjustments (e.g., De Bruin et al., 2010; Benoit et al., 2014), specially synthesized musical tracks (e.g., Cancela et al., 2014), or augmented pre-existing music to highlight beat events (e.g., Thaut et al., 1996). This research has demonstrated benefits of music as a stimulus for gait improvement, and the benefits of music can exceed those of metronomic cues (Wittwer et al., 2013). However, interpretations of such benefits are often couched in the above-described model of SMS; walking to music is taken to involve matching step events to the down-beats or “pulse” within the music. Hence, music is considered a special example of beat-based cueing, a “*metronome+*.” It is still considered essentially the presentation of time intervals, but with better signposted interval boundaries (being embedded in the musical structure), and/or being a metronome which is emotionally engaging or motivational. This view still makes it hard to understand why music as gait-cue can lead to spatial / velocity benefits (Wittwer et al., 2013) when compared to metronome cueing. A view of music that takes into account the action-relevance of musical sounds may be more explanatory.

Music is a complex and highly structured concatenation of events, the result of skillfully sequenced actions by musicians. As such, music contains multiple, nested opportunities for action which include mapping movement event boundaries to down beats, but may also include other cues to movement. Music is rarely discrete, but rather a continuous stream of sound sculpted across different time-scales to include edges (e.g., beats), contours (e.g., melodies) and landscapes (e.g., chord progressions). Different parameters of continuous gait cycles may map to these different events in the music, not necessarily just heel-strike to down beat. This affords flexibility to adapt the ongoing gait pattern to the auditory events, without the rigidity of a one-to-one mapping implied in metronomic cueing. For example, a patient may either lift his/her toe off with a beat, place the heel with a chord, or swing the leg with part of a melody, and still have the veridical experience of being in time with the sound. They may also switch unconsciously between these mappings mid-walk, and still be adaptively matching the walking pattern to the sound, such is the nested nature of multiple musical events. Hence, music continuously provides multiple opportunities within each cycle to prospectively map ongoing movements to. Moreover, music is often described as conveying a sense of motion above the individual auditory events that comprise it (Eitan and Granot, 2006; Zhou et al., 2015). This may afford a mapping for the experience of forward propulsion that continuous walking generates, which in turn may explain why walking to music can lead to greater overall gait velocity than a metronome of the same tempo (Wittwer et al., 2013). These possibilities indicate the importance of conceptualizing music as cuing more than step intervals.

The promise of music as a special form of gait-cueing for PD is further enhanced by the creative nature of music in that many parameters of musical sound may be sculpted and composed to afford desired actions. With a metronome, tempo and interval variance (e.g., 1/f noise) are about the only degrees of freedom available to the experimenter/clinician, but within the constraints of a particular tempo music is free to vary in a multitude of ways. Harmonic structure of chords, and balance between bass and melody have been shown to have influences on movement (Hove et al., 2014; Komeilipoor et al., 2015). Stylistic choices, such as instrumentation, may be adapted to suit the listener's musical preferences, with the intention of enhancing *the invitations to action afforded* (Whitagen et al., 2012; Schiavio and Altenmüller, 2015). Moreover, composing music for walking allows auditory affordances to be adapted to the capabilities of the listener-walker, which may be particularly relevant for PD (Cancela et al., 2014). Given the pre-existing challenges for PD patients to generate “healthy” styles of gait, one can imagine compositions of music which affords cyclical step patterns and forward propulsion, but without the regimentation of marching music, for example. Such suggestions are speculative, but are warranted within the proposed framework as an underexplored approach to musical action sounds for gait cueing.

## Conclusion

Our opinion is that by considering the affordances that auditory events such as footstep sounds or music specify for action, that go beyond the metronomic indication of temporal intervals, auditory cues for gait in PD can be better understood and further expanded. It is important to recognize that this view is not in opposition to existing approaches, but rather offers a reinterpretation of previous findings. Metronomes may afford intercepting a regular beat (or reacting to it) through movement, while action sounds and music afford this and more. Moreover, these opportunities are mediated by the actor's capabilities to detect and act on such information in the sound, which should inform the tailoring of cues to the characteristics of people with PD. By considering auditory cues for walking in PD along with the mapping of action capabilities to temporally patterned sound events, a richer, more flexible understanding and development of cueing approaches to gait in Parkinson's is possible.

## Author contributions

Both authors MR and CC conceived of the ideas presented in the manuscript, MR wrote the main draft which CC edited critically to revise intellectual content. Both authors approve the final manuscript and agree to take responsibility for content contained.

### Conflict of interest statement

The authors declare that the research was conducted in the absence of any commercial or financial relationships that could be construed as a potential conflict of interest.
